# The regulatory network of *Coprinopsis cinerea* transcription factor Skn7 collaborates with bHLH1 during fungal-fungal interactions

**DOI:** 10.1128/spectrum.00484-25

**Published:** 2025-07-30

**Authors:** Huifang Zhao, Na Pang, Xinyue Meng, Qiuyu Qian, Qiqi Han, Qun Han, Xinlei Zhang, Zemin Fang, Juanjuan Liu

**Affiliations:** 1 School of Life Sciences, Anhui University 428675 , Hefei, Anhui, China; 2 Anhui Key Laboratory of Biocatalysis and Modern Biomanufacturing, Hefei, Anhui, China; 3 Anhui Provincial Engineering Technology Research Center of Microorganisms and Biocatalysis, Hefei, Anhui, China; Jagiellonian University, Kraków, Poland

**Keywords:** fungal-fungal interaction, defense response, Skn7, bHLH, ChIP-seq

## Abstract

Fungal-fungal interactions have attracted increasing attention due to alterations in physiological and developmental patterns and the upregulation of secondary metabolites. However, the molecular mechanisms underlying this phenomenon remain poorly understood. Previously, we reported that intracellular reactive oxygen species (ROS) function as signal molecules to induce defense responses in a basidiomycete fungus, *Coprinopsis cinerea*, against a Mucoromycete fungus *Gongronella butleri* w5, concomitant with the upregulation of Skn7, a regulator participating in oxidative stress response. In this study, gene silencing and overexpression experiments demonstrated the involvement of Skn7 in mycelial growth and antioxidation in *C. cinerea* during cocultivation. According to the transcriptomic data, Skn7 was associated with the expression profiles of intracellular antioxidative enzymes, laccases, and secondary metabolite biosynthesis genes, including *easA* and *nrps*. Chromatin immunoprecipitation followed by massively parallel sequencing analysis, along with electrophoretic mobility shift assay experiments, further confirmed the direct binding of Skn7 to these gene promoters. Another transcription factor, bHLH1, was identified to directly interact with Skn7 in regulating antioxidative defense mechanisms without influencing the expression of genes involved in secondary metabolite biosynthesis. Our work demonstrates Skn7 as a crucial transcription factor that orchestrates the regulatory network and targets downstream genes, thereby protecting *C. cinerea* against extracellular oxidative stress and elevated intracellular ROS levels during fungal-fungal interactions. Furthermore, we reveal the collaborative role of bHLH1 and Skn7 in fungal antagonism defense.

**IMPORTANCE:**

Fungal-fungal interactions are widespread and play a significant role in the function and stability of ecosystems. This study reveals the molecular mechanisms by which *Coprinopsis cinerea* employs the transcription factors Skn7 and bHLH1 to coordinately regulate antioxidant defense mechanisms during its antagonistic interaction with *Gongronella butleri* w5. Skn7 not only directly regulates the expression of intracellular antioxidative enzymes and laccases but also regulates secondary metabolite biosynthesis genes. The two transcription factors collaborate to protect *C. cinerea* against oxidative stress. These findings deepen our understanding of signal transduction and defense mechanisms during fungal interactions, as well as provide new insights into the regulation of secondary metabolites in fungi.

## INTRODUCTION

In the soil, fungi account for approximately 75% of the total biomass of soil microorganisms (1). Fungi extend their mycelia to acquire new nutrients and territories, inevitably coming into contact with and antagonizing other microorganisms (2). These interactions play a crucial role in determining microbial species and abundance, driving microbial community evolution, and ultimately establishing a stable metabolic cycle in soil (3–5). Changes in fungal mycelial morphology, as well as physiological and developmental patterns, are frequently observed during fungal interactions (6). To maintain intracellular homeostasis, fungi need to regulate metabolic pathways, synthesize attack- and defense-related metabolites, and release extracellular enzymes in response to environmental changes (7–9). It is widely acknowledged that fungal coculture in the laboratory provides a feasible and efficient strategy for simulating natural fungal interactions (10, 11). Such cocultures can stimulate the synthesis of diverse secondary metabolites and enzymes, including terpenoids, alkaloids, polyketides, and laccases, thus exhibiting great potential applications (7, 10, 12–19).

Chemical signals, enzymes, or related proteins produced by cocultured microorganisms have been reported to mediate fungal interactions. For example, *p*-hydroxybenzoic acid isolated from *Gongronella butleri* w5 and *β*-carotene produced by *Rhodotorula mucilaginosa* induce upregulation of laccases in *Coprinopsis cinerea* and *Pleurotus eryngii* var. *ferulae*, respectively (10, 20, 21). Nonribosomal peptide synthetase (NRPS)-derived isoquinolines and lipopeptides produced by *Ralstonia solanacearum* mediate its antagonism with the plant-pathogenic fungus *Aspergillus flavus* (22). These compounds can lead to significant oxidative stress in fungi, resulting in increased intracellular reactive oxidative species (ROS) (22–26). ROS consist of both free radical oxygen intermediates and non-free radicals such as ∙O_2_, OH-, and H_2_O_2_, playing critical roles in signal transduction processes. They are associated with enhancing superoxide dismutase (SOD) activity, spore germination, and mycelial growth during fungal cocultivation (22, 27–29). At the transcriptional level, two regulatory proteins, including a member of the histone acetyltransferase SAGA/ADA complex, GcnE, and a velvet complex member, VeA, as well as an Mby-like transcription factor, BasR, are shown to regulate fungal metabolism during fungal-bacterial communication (30–33). However, the detailed mechanisms underlying silent gene activation during fungal-fungal interactions remain unknown, except for the recently reported VeA1 controlling secondary metabolism production in *Aspergillus nidulans* (7).

Skn7 is a highly conserved fungal heat shock factor-type transcriptional regulator with a heat shock factor (HSF)-type DNA-binding domain (DBD) (34). It has been demonstrated to participate in regulating the response to osmotic/oxidative stress as part of a two-component regulatory system in some Ascomycota fungi, responding to upstream signals and controlling the expression of downstream antioxidant proteins (35). Examples include *Candida albicans* and *Candida glabrata*, where Skn7 regulates *trx2* (encoding thioredoxin), *trr1* (encoding thioredoxin reductase), *tsa1* (encoding thioredoxin peroxidase), and *cat1* (encoding catalase); *Saccharomyces cerevisiae*, where it controls *trx2*, *gpx2* (encoding glutathione peroxidase), *cat1*, *sod1,* or *sod2*; and *A. nidulans* affecting *cat* expression (34, 36–38). Additionally, changes in Skn7 transcriptional levels also lead to abnormal mycelial morphogenesis and development. Overexpression of *skn7* induces mycelial formation, while its inactivation results in filamentation defects in *C. albicans* (39). Similarly, the deletion of *skn7* affects both asexual and sexual development in *A. flavus,* while interference with *skn7* inhibits mycelial growth in *Ganoderma lucidum* (40, 41). Based on key residue mutations observed in *C. albicans*, it appears that the regulation of Skn7 on morphogenesis is independent of its response to oxidative stress (34). Furthermore, the recent discovery of Skn7’s functions, including its regulatory role in triterpene biosynthesis in *G. lucidum*, as well as its involvement in mycosporine and sterol production in *Xanthophyllomyces dendrorhous*, reveals additional roles of Skn7 in fungi (40, 42).

*C. cinerea* is a basidiomycete fungus distributed worldwide and grows on various substrates (43). It is also known as a model in mushroom developmental biology (44). *G. butleri* w5 is a mucoromycete fungus isolated from topsoil enriched with decomposing leaves and branches (45). This species is distributed all over the world, occurring at moderate frequencies in subtropical regions and warm climates (46). It has been proven to interact with plants and microorganisms, facilitating the degradation of complex carbon sources and influencing soil nutrient cycling (47, 48). Previously, we demonstrated that the intracellular ROS acted as signal molecules to stimulate defense responses by *C. cinerea* during interaction with *G. butleri* w5, among which laccase Lcc9 was triggered to take part in defense (19). Through transcriptomic and quantitative RT-PCR (qRT-PCR) analysis, a transcription factor with relatively high sequence similarity to *S. cerevisiae* Skn7 was found to be highly expressed during coculture, but its expression was inhibited by ROS scavenger treatment. This protein was speculated to transmit the ROS signal and regulate gene transcription. Our study here reveals the essential role of Skn7 in *C. cinerea* during its confrontation with *G. butleri* w5, particularly in controlling antioxidative response, promoting mycelial growth, and regulating the expression of secondary metabolite biosynthesis genes. Furthermore, we demonstrate that a newly identified interacting transcription factor, bHLH1, cooperates with Skn7 to transduce signals and function in fungal-fungal interactions. The basic helix-loop-helix (bHLH) transcription factors, consisting of an N-terminal basic region and a C-terminal HLH region, are widely conserved in eukaryotes and function as transcriptional activators and/or repressors of genes involved in diverse biological processes, including stress responses and development (49, 50). The basic region typically recognizes the E-box (CANNTG) or G-box (CACGTG), while the HLH region mediates homo- and/or heterodimerization, enabling the basic region to bind to target DNA *cis*-regulatory sequences (51). However, there are few reports on bHLH transcription factors in fungi, especially basidiomycete fungi.

## RESULTS

### Skn7 is critical for mycelial growth, oidia production, and the response to oxidative stress in *C. cinerea* during interspecific interactions

**Fig 1:**
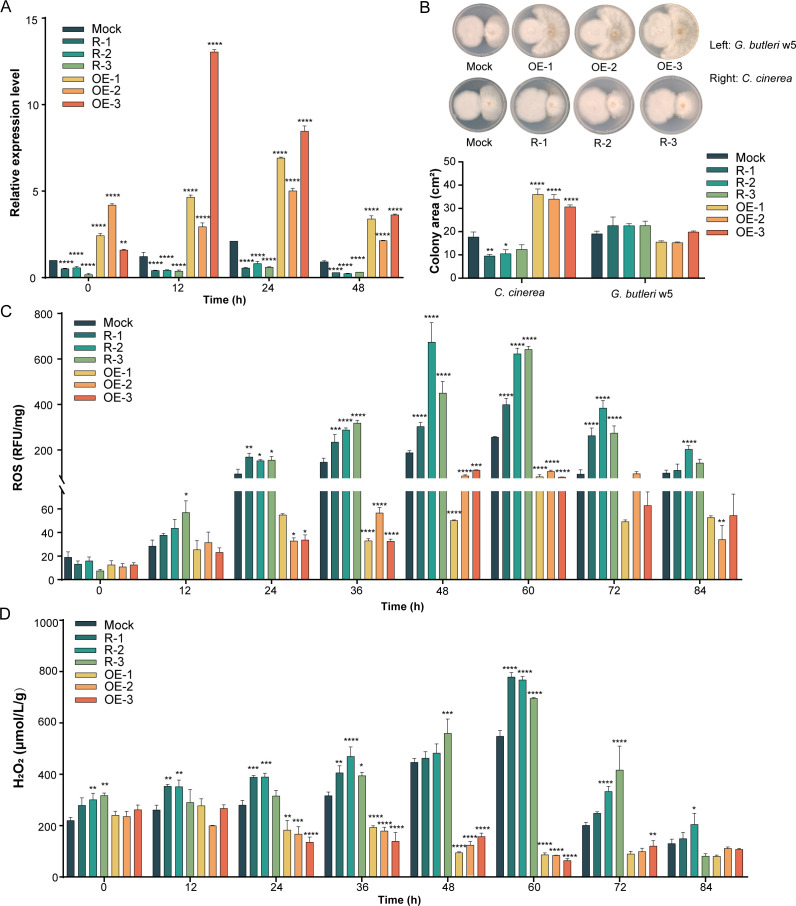
Skn7 is critical for mycelial morphology and the response to oxidative stress in *C. cinerea* during interspecific interactions with *G. butleri* w5. (**A**) The transcriptional levels of *skn7* in *skn7*-overexpressed and *skn7*-silenced transformants. The *skn7* transcript in the Mock transformant at 0 h of liquid cocultivation was set as the baseline. (**B**) The colony area of the seven clones under coculture with *G. butleri* w5 on plates. (**C and D**) ROS (**C**) and H_2_O_2_ (**D**) concentrations of the seven clones during liquid coculture. Data show mean ± standard deviation, *n* = 3. *P < 0.05, **P < 0.01, ***P < 0.001, and ****P < 0.0001.

*C. cinerea* protoplasts were transformed with either the *skn7*-overexpressed plasmid or the *skn7*-silenced plasmid. Three overexpression and three silencing transformants were randomly chosen for comparison with the Mock transformant to elucidate the role of Skn7 in fungal interspecific interactions. According to qRT-PCR analysis, the transcriptional levels of *skn7* in the three overexpression transformants (named OE-1, OE-2, and OE-3) increased by 2.3–13.1-fold during 0–48 h of coculture with *G. butleri* w5, relative to the Mock transformant (Fig. 1A). In contrast, the transcriptional levels of *skn7* in the three silencing transformants (named R-1, R-2, and R-3) exhibited a significant reduction (*P* < 0.0001; Fig. 1A).

The growth rates of the seven clones were individually assessed in both axenic cultures and cocultures. No significant differences in mycelial growth were observed among all strains when cultured on FAHX agar plates. However, upon exposure to antagonistic interaction with *G. butleri* w5 on SAHX agar plates, the mycelial growth rate of the *skn7*-overexpressed transformants increased more than twofold (Fig. 1B). Conversely, the silencing of *skn7* reversed the sensitivity of *C. cinerea* to *G. butleri* w5, resulting in a 53.5% reduction in mycelial growth rate in *skn7*-silenced transformants compared to the Mock transformant (Fig. 1B). The absolute number of oidia showed a trend consistent with the growth phenotype. It was much higher in *skn7*-overexpressed transformants (average number = 2.1 × 10^7^) but significantly lower in *skn7*-silenced transformants (4.2 × 10^6^; *P* < 0.001) than that in the Mock transformant (1.6 × 10^7^) following a 5-day interaction with *G. butleri* w5. Furthermore, an upregulated formation of hyphal side branches was observed after *skn7* overexpression, whereas it decreased upon *skn7* silencing (Fig. S1).

The intracellular ROS and H_2_O_2_ concentrations in *C. cinerea* during fungal interactions were further compared. As exemplified by 60 h of coculture, overexpression of *skn7* led to an approximately 59%–69% reduction in ROS concentrations and around 85% reduction in H_2_O_2_ concentrations, whereas silencing of skn7 resulted in a 156%–250% increase in ROS concentrations and a slight increase in H_2_O_2_ concentrations (Fig. 1C and D; Table S1-1). These results highlight the critical roles of *C. cinerea* Skn7 in mycelial growth, oidia production, and the response to oxidative stress during fungal-fungal interactions.

### Skn7 regulates the transcription of intracellular antioxidative enzymes, laccases, and secondary metabolite biosynthesis genes

**Fig 2:**
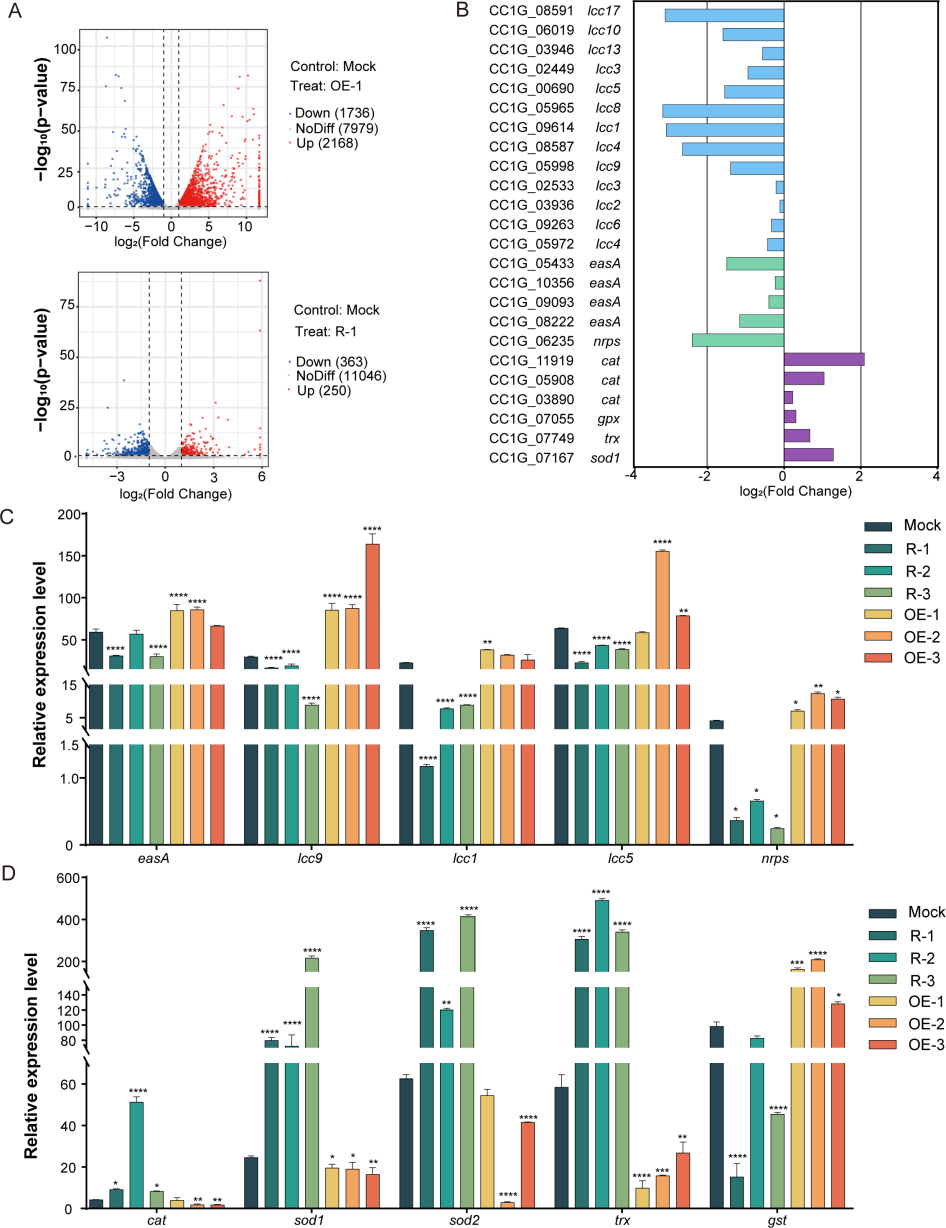
Skn7 regulates the transcription of key enzymes involved in oxidative stress confrontation and secondary metabolite biosynthesis. (**A**) Cluster analysis of DEGs in transformants OE-1 and R-1 relative to the Mock transformant. (**B**) The transcriptional levels of laccase isoenzyme genes (in blue) and key genes participating in intracellular oxidative stress confrontation (*cat*, *gpx*, *trx*, and *sod1* in purple) and secondary metabolite biosynthesis (*easA* and *nrps* in green) in transformant R-1 relative to the Mock transformant according to the transcriptomic data. (**C and D**) qRT-PCR analysis of the transcriptional levels of *cat*, *sod1*, *sod2*, *trx*, *gst*, *lcc1*, *lcc5*, *lcc9*, *nrps*, and *easA* in *C. cinerea* at 36 h of coculture. The transcripts of each gene in the Mock transformant at 0 h of liquid cocultivation were set as the baselines, respectively. Data show mean ± standard deviation in panel **C and D**, *n* = 3. *P < 0.05, **P < 0.01, ***P < 0.001, and ****P < 0.0001.

The transformants Mock, OE-1, and R-1 were chosen for transcriptomic analysis to investigate the gene transcriptional profiles regulated by Skn7 in *C. cinerea* during its face-off with *G. butleri* w5. There were 2,168 upregulated genes and 1,736 downregulated genes in the *skn7*-overexpressed transformant OE-1, while 250 upregulated genes and 363 downregulated genes were observed in the *skn7*-silenced transformant R-1 (fold change > 1.0; *P* < 0.05; Fig. 2A; Table S1-2 and 3). A total of 321 differentially expressed genes (DEGs) overlapped. Kyoto Encyclopedia of Genes and Genomes (KEGG) enrichment analysis revealed that genes associated with Skn7 expression were significantly enriched in pathways related to signal sensing (including *rcb* [CC1G_15339, CC1G_02151, CC1G_07395, CC1G_02153, and CC1G_02137] encoding pheromone receptor protein), intracellular oxidative stress confrontation (including *sod1* [CC1G_07167], *sod2* [CC1G_03559], *cat* [CC1G_11919], *trx* [CC1G_08103], and *gpx* [CC1G_07055]), and laccases (*lcc1*, *lcc4*, *lcc5*, *lcc5*, *lcc8*, *lcc9*, *lcc10,* and *lcc17*) (52–55) (Fig. 2B; Table S1-4). Furthermore, the expression of two secondary metabolite biosynthesis genes was also enriched. These included *easA* (CC1G_05433 and CC1G_08222), which encoded ergot alkaloid biosynthetic protein A and exhibited alternate activities in various fungi, contributing to the diversification of the ergot alkaloid production pathway, and *nrps* (CC1G_06235), which encoded a non-ribosomal peptide synthetase involved in the production of non-ribosomal peptides that acted as fungal virulent factors (8, 56–61).

The qRT-PCR results were largely consistent with the transcriptome data, revealing that the transcriptional levels of chosen genes, including *gst* (encoding glutathione S-transferase), *lcc1*, *lcc5*, *lcc9*, *easA* (CC1G_08222), and *nrps* were upregulated in three *skn7*-overexpressed transformants but downregulated in three *skn7*-silenced transformants relative to the Mock transformant (Fig. 2C). However, the transcripts of *sod1*, *sod2*, *cat*, and *trx* were reduced following *skn7* overexpression and elevated upon *skn7* silencing. Notably, despite *gst* (CC1G_11081) exhibiting a fold-change value of 2.1 in OE-1 and not aligning with R-1 in the sequencing data, its transcriptional levels were dramatically increased at 36 h of coculture post-*skn7* overexpression, while they were significantly decreased following *skn7* silencing (*P* < 0.0001; Fig. 2D). Taken together, these transcriptional profiles suggest a critical reprogramming in oxidative stress response and metabolism upon *skn7* overexpression or silencing.

### Skn7 differentially regulates antioxidative enzymatic activities in cocultivated *C. cinerea*

**Fig 3:**
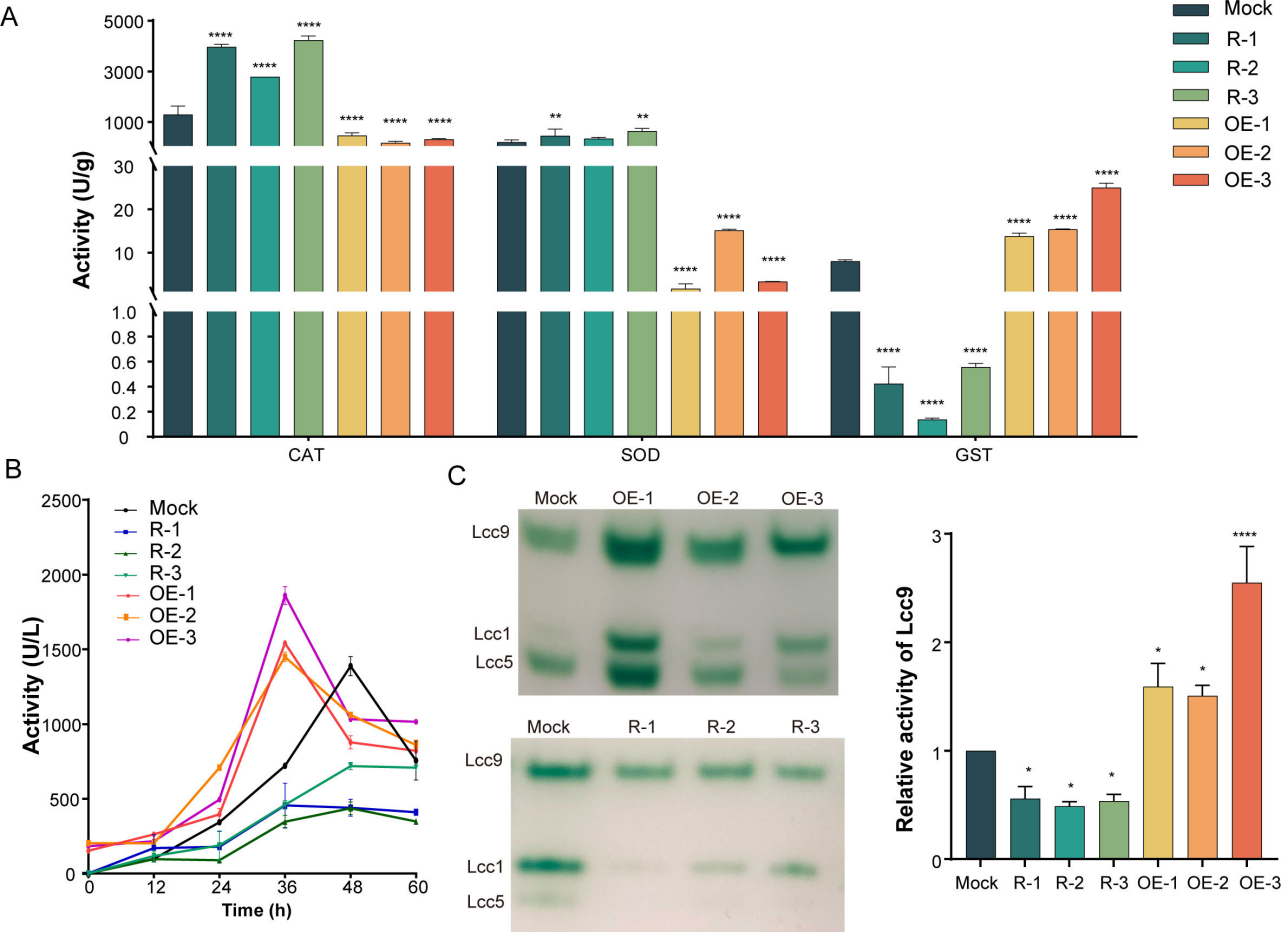
Skn7 differentially regulates antioxidative enzyme activities in cocultivated *C. cinerea*. (**A**) CAT, SOD, and GST activities in *skn7*-overexpressed and *skn7*-silenced transformants at 60 h of liquid cocultivation. (**B and C**) The laccase activity in *skn7*-overexpressed (**B**) and *skn7*-silenced (**C**) transformants during liquid cocultivation. The quantification data show mean ± standard deviation, *n* = 3. *P < 0.05, **P < 0.01, and ****P < 0.0001.

The specific activities of SOD, CAT, and GST in *C. cinerea* were measured at 60 h of cocultivation to assess their expressional levels (Fig. 3; Table S1-1). Compared to the Mock transformant, the three overexpressing transformants showed a 64%–87% reduction in CAT activity, a 92%–99% decrease in SOD activity, and a 172%–312% increase in GST activity (Fig. 3A). In contrast, in the three silencing transformants, there was a 216%–328% increase in CAT activity, a 174%–357% increase in SOD activity, and a 93%–98% decrease in GST activity. These findings suggest that Skn7 positively regulates the expression of GST while negatively affecting the expression of CAT and SOD in *C. cinerea* during interspecific interaction with *G. butleri* w5.

Furthermore, laccase activity and isozyme identification through native polyacrylamide electrophoresis (native-PAGE) analysis confirmed the positive regulatory role of Skn7 on the expression of Lcc1, Lcc5, and Lcc9 (Fig. 3B and C). Overexpression of Skn7 not only enhanced laccase production but also led to an earlier peak in activity and sustained relatively high activity for a prolonged duration compared to the Mock transformant. Specifically, the maximum total activity reached 1,541, 1,450, and 1,860 U/L in OE-1, OE-2, and OE-3, respectively, at 36 h of coculture, as opposed to the maximum activity of 1,388 U/L in Mock at 48 h of coculture. Moreover, the maximum activity in three *skn7*-silenced transformants ranged from 440 to 720 U/L. Biomass detection at 48 h of coculture revealed a marginal reduction in *skn7*-silenced transformants, while overexpression of *skn7* resulted in a slight increase in biomass relative to the Mock transformant (Fig. S2).

### Genes involved in oxidative stress defense and secondary metabolite biosynthesis are direct targets of Skn7

**Fig 4:**
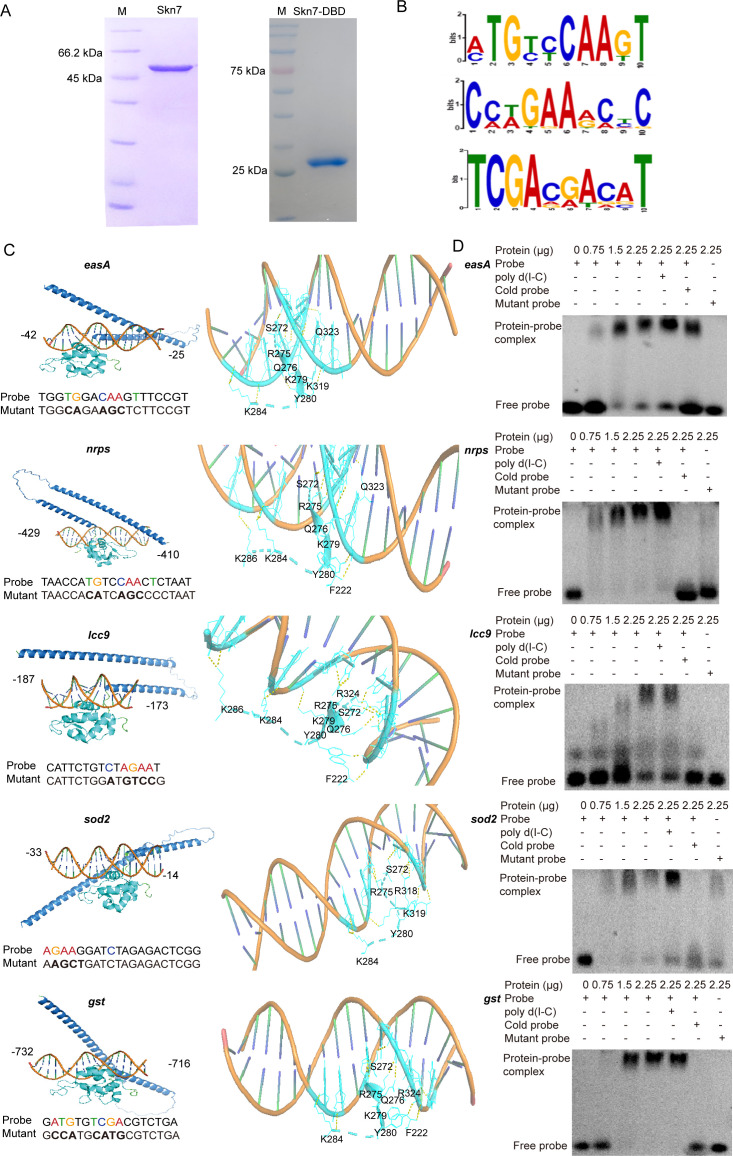
Skn7 directly binds to the promoter regions of genes involved in oxidative stress defense and secondary metabolite biosynthesis. (**A**) The full-length Skn7 protein and Skn7-DBD protein were heterologously expressed and purified. (**B**) The DNA binding motifs of Skn7 according to ChIP-seq data. (**C**) Structure modeling of the interaction between Skn7 and DNA fragments located in the promoter regions of *nrps*, *easA*, *lcc9*, *sod2*, and *gst*. The mutations introduced at the conserved nucleotide sites were highlighted in color. (**D**) Electrophoretic mobility shift assays verified the direct binding of Skn7 to the promoter regions of *nrps*, *easA*, *lcc9*, *sod2*, and *gst*.

Chromatin immunoprecipitation followed by massively parallel sequencing (ChIP-seq) analysis was performed to investigate whether Skn7 directly participated in the transcriptional regulation of detoxification enzymes and secondary metabolite biosynthesis genes. The soluble full-length Skn7 protein (Fig. 4A) was used as an antigen to immunize New Zealand White rabbits to produce the specific antibody against Skn7 (anti-Skn7). Three independent ChIP-seq experiments on the wild-type (WT) parent *C. cinerea* mycelia in coculture identified a total of 351 peaks (121, 81, and 149, respectively) corresponding to 199 genes (70, 34, and 95, respectively) (Table S1-5). The peak intensity map derived from the ChIP-seq data revealed a significant enrichment of Skn7 binding to specific promoter regions of *C. cinerea* when cocultured with *G. butleri* w5. According to the MEME analysis of 1 kb 5′-upstream regions of Skn7-targeted genes, three motifs, including TGYYCAA (motif 1, *Y* = C/T), NWGAANN (motif 2, *N* = A/C/T/G, *W* = A/T), and TCGAVRW (motif 3, *V* = G/A/C, *R* = A/G), were identified (Fig. 4B). Notably, the promoter regions of *nrps* (bp −429 to −410) and *easA* (bp −42 to −25) contained motif 1; *lcc9* (bp −187 to −173) and *sod2* (bp −33 to −14) harbored motif 2; and *gst* (bp −732 to −716) possessed motif 3.

AlphaFold3 was used to simulate the interaction between Skn7 and the DNA fragments obtained above (Fig. 4C). It was observed that several amino acids of Skn7 formed stable hydrogen bonds with the five promoter region sequences. Although the specific interaction amino acids varied across the five models, four amino acids—S272, R275, Y280, and K284—were consistently conserved, which supported the critical role of the DBD (222–319 aa) in binding. Additionally, two other amino acids adjacent to the DBD, Q323 and R324, were inferred to participate in the interaction.

Electrophoretic mobility shift assays (EMSAs) were conducted to verify the direct interaction between Skn7 and these DNA probes. As shown in Fig. 4D, the bindings were specific, as the addition of poly d(I-C) did not affect the formation of shifted bands. Conversely, the inclusion of a 10-fold excess of unlabeled DNA fragments inhibited the formation of the chemiluminescent complexes (Fig. 4D). Additionally, random mutations introduced at the conserved sites highlighted in color (Fig. 4C) abolished the protein’s ability to bind to the probes, suggesting that Skn7 specifically recognizes the motif sequences identified through ChIP experiments (Fig. 4D). Collectively, these results provide evidence for the direct regulatory role of Skn7 in oxidative stress defense and secondary metabolite biosynthesis.

### bHLH1 interacts with Skn7 to function in fungal-fungal interactions

Nuclear proteins from *C. cinerea* cocultured with *G. butleri* w5 were extracted, and co-immunoprecipitation (Co-IP) was performed to bait the interacting partners of Skn7 (Table S1-6). One protein, designated as CC1G_05514, which harbored a characteristic bHLH domain, was identified and named bHLH1 through ESI-LC-MS/MS analysis. The bHLH1 gene was 3,186 bp in length and encoded a 1,061-amino-acid protein. Sequence alignment revealed that bHLH1 shared the highest sequence similarity with the homolog from *Candolleomyces aberdarensis* (66%). Its bHLH domain displayed 32% and 33% identity with *C. albicans* Cph2p and *Aspergillus fumigatus* SrbA, respectively (Fig. S3). Furthermore, the 120-aa-length bHLH domain comprised a basic region of 33 aa and two helices (9 aa each) that were connected by a 42-aa loop.

**Fig 5:**
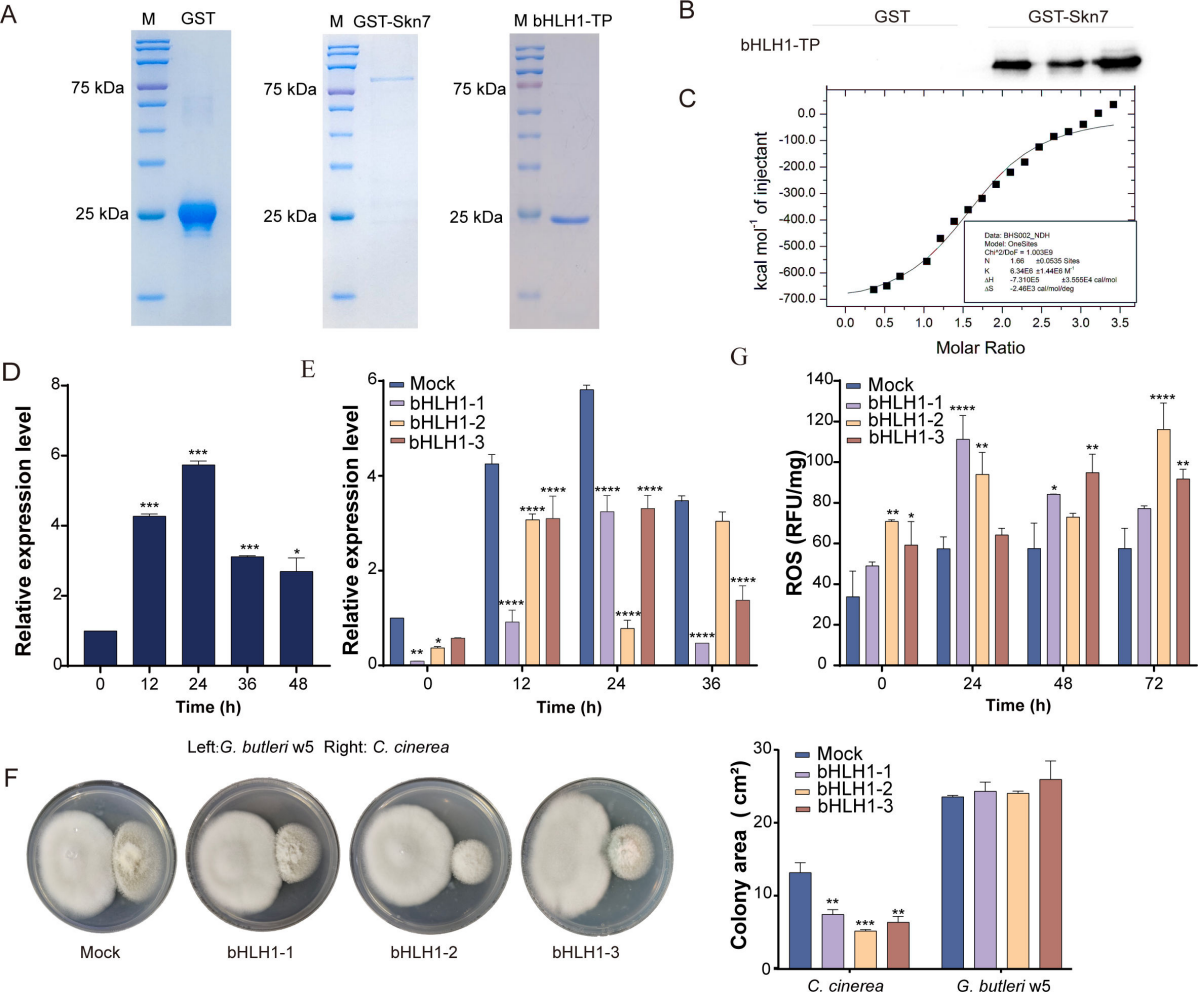
bHLH1 interacts directly with Skn7 in *C. cinerea* to participate in defense against *G. butleri* w5. (**A**) Heterologous expression and purification of the truncated protein of bHLH1, GST tag, and GST-Skn7. (**B and C**) *In vitro* pull-down and ITC verification of the interaction between Skn7 and bHLH1. (**D and E**) The transcriptional levels of *bHLH1* in WT parent (**D**) and *bHLH1*-silenced (**E**) *C. cinerea* during liquid coculture with *G. butleri* w5. The *bHLH1* transcript in the WT or Mock transformant at 0 h of cocultivation was set as the baseline. (**F**) The colony area of Mock and *bHLH1*-silenced transformants under coculture with *G. butleri* w5 on plates. (**G**) ROS concentrations in Mock and *bHLH1*-silenced transformants during liquid coculture. Data show mean ± standard deviation in panels D–G, *n* = 3. *P < 0.05, **P < 0.01, ***P < 0.001, and ****P < 0.0001.

Despite multiple attempts, the soluble full-length bHLH1 could not be successfully obtained. Consequently, a truncated form of bHLH1, containing the domain predicted to interact with Skn7 via HDOCK simulation (Fig. S4), was heterologously expressed and purified (Fig. 5A). The direct interaction between bHLH1 and Skn7 was confirmed using an *in vitro* pull-down assay (Fig. 5B). The binding affinity was further characterized by isothermal titration calorimetry (ITC) assay, exhibiting a dissociation constant (KD) value of approximately 6.34 µM (Fig. 5C).

qRT-PCR analysis showed that the transcriptional level of *bHLH1* was strongly induced in *C. cinerea* under coculture conditions, reaching its peak at 24 h post-coculture (Fig. 5D). To investigate the role of bHLH1 in interspecific interactions, *bHLH1*-silenced transformants were successfully constructed. Three transformants, named bHLH1-1, bHLH1-2, and bHLH1-3, exhibited a 50%–90% reduction in *bHLH1* transcriptional levels during 0–36 h of coculture (Fig. 5E). Compared to the Mock transformant, *bHLH1* silencing led to a significant decrease in the mycelial growth rate of *C. cinerea* when cocultured with *G. butleri* w5 (*P* < 0.05 and *P* < 0.001; Fig. 5F). Light microscopy observations indicated that *bHLH1* silencing resulted in decreased mycelial density and increased formation of hyphal side branches (Fig. S5). Additionally, the ROS concentrations in *bHLH1*-silenced transformants were markedly higher than those in the Mock transformant (Fig. 5G). These results demonstrate that bHLH1 interacts with Skn7 to play a crucial role in *C. cinerea* during its antagonistic interaction with *G. butleri* w5.

### bHLH1 regulates the transcription of genes associated with oxidative stress defense

**Fig 6:**
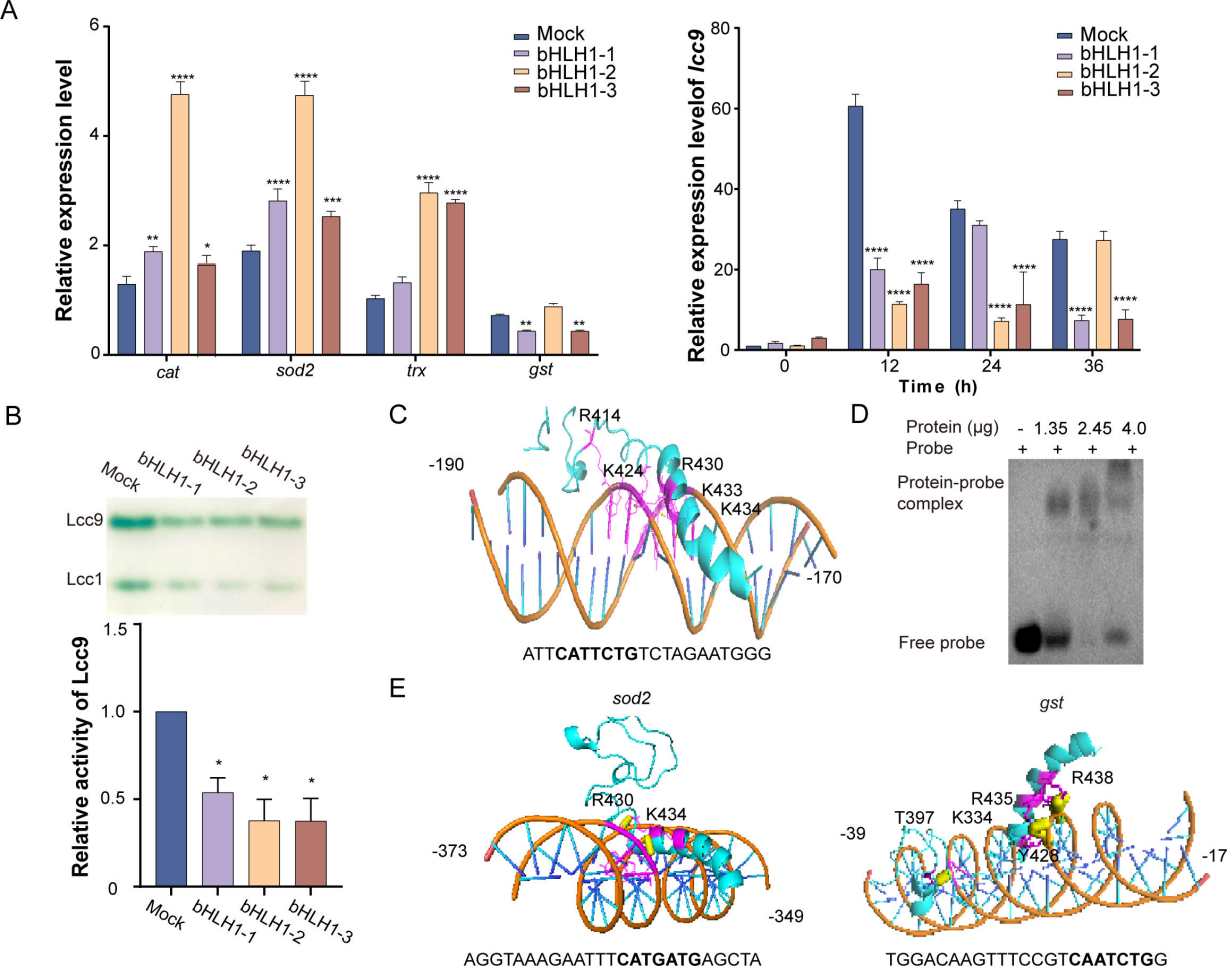
bHLH1 directly regulates the transcription of genes associated with oxidative stress defense during fungal-fungal interactions. (**A**) qRT-PCR analysis of the transcriptional levels of *cat*, *sod2*, *trx*, *gst*, and *lcc9* in Mock and *bHLH1*-silenced *C. cinerea* at 36 or 0–36 h of liquid coculture. (**B**) Quantification of Lcc9 activities. (**C and D**) Structure modeling and EMSA verification of the direct interaction between bHLH1 and the *lcc9* promoter. (**E**) Structure modeling of the interaction of bHLH1 with the *sod2* or *gst* promoter. Data show mean ± standard deviation in panels **A** and **B**, *n* = 3. *P < 0.05, **P < 0.01, ***P < 0.001, and ****P < 0.0001.

To investigate whether bHLH1 can regulate the expression of genes controlled by Skn7 as mentioned above, the transcriptional levels of *cat*, *trx*, *sod2*, *gst*, *lcc9*, *nrrps,* and *easA* were detected. During coculture with *G. butleri* w5, three *bHLH1*-silenced transformants exhibited upregulated transcripts of *cat*, *trx*, and *sod2* compared to the Mock transformant. In contrast, downregulated transcripts of *gst* and *lcc9* were observed (Fig. 6A). However, the transcriptional levels of *nrps* and *easA* showed no significant change (Fig. S6). Lcc9 was taken as an example for further study. The laccase activity revealed a significant decrease in the expression of Lcc9 after *bHLH1* silencing (Fig. 6B). AlphaFold3 simulation and EMSA assay confirmed the direct binding of truncated bHLH1 to the promoter region of *lcc9* (bp −190 to −170) (Fig. 6C and D). Furthermore, the DNA fragments in the promoters of *gst* (bp −39 to −17) and *sod2* (bp −373 to −349) were also predicted to interact directly with bHLH1 (Fig. 6E).

The transcriptional levels of *skn7* were further detected in both the Mock and *bHLH1*-silenced transformants. They were observed to be comparable across all four clones. Additionally, the transcriptional levels of *bHLH1* remained largely unchanged following either overexpression or silencing of *skn7* (Fig. S7). According to the ChIP-seq data, bHLH1 was not a direct target of Skn7. Thus, while Skn7 and bHLH1 are involved in fungal-fungal interactions as cooperators, they function independently of each other.

## DISCUSSION

Skn7 is a critical transcription factor that is ubiquitously present in fungi. It plays an essential role in facilitating fungal responses to external stress, modulating the expression of genes associated with oxidative stress resistance, and maintaining cellular homeostasis (52). As research on Skn7 continues to advance, its multifaceted functions in fungal biology are becoming increasingly apparent. These include involvement in morphological development, cell wall reorganization, fungicide sensitivity, osmotic stress adaptation, secondary metabolite production, and cellular differentiation (41, 62–65). The majority of existing evidence pertains to yeasts and pathogenic or opportunistic fungi such as *S. cerevisiae*, *C. albicans*, *C. glabrata*, *X. dendrorhous*, *Cryptococcus neoformans*, *A. flavus*, and *A. fumigatus* (52, 64, 66–68). However, the physiological function and functional interactions of Skn7 in other fungi remain unexplored. In this study, we employed a combination of gene silencing and overexpression experiments, RNA-seq, ChIP-seq, Co-IP, and EMSA assays to elucidate the cooperative function of Skn7 and bHLH1 in the basidiomycete *C. cinerea* during fungal-fungal interactions. Specifically, they regulated the expression of intracellular antioxidative enzymes and laccases, thereby mediating the defense mechanisms. Furthermore, Skn7 was also involved in the regulation of gene expression related to secondary metabolite biosynthesis.

*C. cinerea* Skn7 features an HSF-type DBD and a receiver domain. It also harbors a leucine-zipper motif similar to that of *C. albicans* Skn7 but distinct from the motif in *S. cerevisiae* Skn7 (34, 52) (Fig. S8). Transcript profiling and ChIP-seq analysis revealed that Skn7 is directly bound to the promoters of some intracellular antioxidative enzyme genes and *lcc9,* serving as a defense strategy in *C. cinerea* during the confrontation. This finding aligned with Skn7’s established role in other fungi and supported our hypothesis that Skn7 transmitted the ROS signals derived from *G. butleri* w5 into the nucleus (19). Although the possibility that multiple plasmid copies were introduced into the cells and led to different gene expression levels among three *skn7*-overexpressed or *skn7*-silenced transformants, the antioxidative enzymes exhibited contrasting transcriptional profiles upon *skn7* overexpression or silencing (Fig. 2C). Specifically, Skn7 positively regulated the expression of GST and Lcc9 while negatively regulating CAT and SOD (Fig. 3). Although most oxidative stress-responsive genes were upregulated in *C. cinerea* during interaction with *G. butleri* w5, the increased expression of Lcc9 and GST might contribute to eliminating oxidative stress dependent on Skn7. Lcc9, secreted extracellularly by *C. cinerea*, oxidizes or transforms most toxic metabolites (benzene and naphthalene compounds) from *G. butleri* w5 (19, 20). Other toxic metabolites that enter cells may be conjugated with glutathione by GST, according to its detoxification role against organic compounds in many fungi (69, 70).

Skn7 is associated with the transcription of oxidative stress-responsive genes; however, the use of diverse background strains and varying oxidative stress conditions has introduced interpretative challenges to both *in vivo* and *in vitro* observations. For example, deletion of *skn7* in yeast increases sensitivity to a broad spectrum of oxidizing agents and reduces the expression of detoxification genes such as *trx2*, *gpx2*, *ctt1*, *sod1*, or *sod2* (36, 71, 72). Conversely, the deletion of FoSkn7 in *Fusarium oxysporum* f. sp. *cubense* negatively affects the expression of many genes encoding ROS-detoxifying enzymes and heat-shock proteins (73). Here, upon *skn7* overexpression, the massive expression of laccases and GST might consume an adequate amount of energy, restricting the synthesis of other intracellular antioxidative enzymes. These enzymes cooperated at a dynamic balance to maintain the homeostasis of fungi. Moreover, fungal responses to oxidative stress are complex and multilayered, involving multiple regulatory cascades, such as HOG and Yap1 pathways (74). Further characterization of additional regulators is necessary to elucidate the intricate signaling networks that collectively determine gene expression in fungal-fungal interactions.

Transcription factors form an intricate network within cells. Notably, oxidized Yap1 has been identified as a transcription factor that collaborates with Skn7 to form a complex on the promoters of certain antioxidative genes (74). In *C. albicans*, motif analysis of ChIP-seq data enriched in Skn7-bound regions reveals consensus binding sites for Efg1 and Ndt80, suggesting potential cooperation between Skn7 and these factors in regulating morphogenesis (34). Through Co-IP, ITC, EMSA, and transcriptional profiling, a novel transcription factor, bHLH1, was discovered to interact with Skn7, directly modulating the expression of genes functioning in oxidative stress response, such as *gst*, *sod2*, and *lcc9,* which harbor the *cis*-regulatory sequence of CANNNTG in their promoters (Fig. 6). Research on bHLH-type transcription factors has predominantly focused on plants and *Aspergillus* species. For instance, bHLH transcription factors act as positive regulators of ROS scavenging, enhancing plant resistance to water stress or *Phytophthora sojae* root rot (75, 76). In *A. fumigatus*, deletion of bHLH homologs DevR and EcdR leads to impaired conidia production, pigmentation, and virulence (77, 78). Furthermore, bHLH participates in the regulation of gene expression encoding antioxidant enzymes against ROS damage in *A. flavus* (79). Given the distinct DNA binding motifs of Skn7 and bHLH1, it is plausible that they can function independently while also interacting synergistically.

Three Skn7-targeted motifs were identified in *C. cinerea* during coculture with *G. butleri* w5. Among them, one harbored a GAA triplet characteristic of heat-shock factor element (80). It was located in promoters of *lcc9* and *sod2*, consistent with our previous conclusion that Lcc9 is an extracellular antioxidative enzyme cooperating with the intracellular defense systems. According to the TFBIND online tool (81) (https://tfbind.hgc.jp/) analysis, a non-canonical TATA box sequence (CCTCTAAACG) was identified at positions bp −71 to −61 in the *sod2* promoter region, with a matching score of 0.764 against the classical TATA box. The binding of Skn7 (bp −33 to −14) upstream of the TATA box might hamper the binding of RNA polymerase or affect the unwinding of DNA, negatively regulating *sod2* transcription. Most transcription factors have a dual role in activating and repressing gene expression (82). The binding region of Skn7 in the *lcc9* promoter (bp −187 to −173) was distant from the TATA box (bp −97 to −92) and served Skn7 as the activator of Lcc9.

Another motif includes a TGYYCAA element located in *nrps* and *easA*, as well as salicylate hydroxylase (*CC1G_01286*) and glucose oxidase (*CC1G_15386*). In *Sclerotinia sclerotiorum*, the SsShy1 (SS1G_02963) plays an important role in the growth, metabolism, and virulence of the fungus, which is affected by salicylic acid (83). Another motif includes a TCGAVRW element located in *gst* and CaMK (*CC1G_02698*) (Table S1-7). In the nematode-trapping fungus *Arthrobotrys oligospora*, five CaMKs are involved in regulating multiple cellular processes, such as growth, tolerance to environmental stresses, conidiation, and virulence formation (84). Furthermore, CaMK1 also plays an important role in the growth, sporulation, and pathogenicity of *Penicillium italicum* (84). The reprogrammed proteins, in response to the upregulation of *skn7* in coculture, collectively function to defend against antagonistic stress. One important aspect to address is that, although the ChIP-seq data identified potential targets for analysis, the consistency among replicates was suboptimal. To ensure the reliability of the findings, the data were further validated through rigorous AlphaFold3 predictions and EMSA results. Multicellular filamentous fungal growth shows significant heterogeneity. During liquid culture, fungal cells at the center of the pellets undergo anaerobic fermentation due to oxygen limitation, whereas cells on the pellet surface are exposed to sufficient oxygen. These microenvironmental differences lead to distinct gene expression patterns and chromatin modification states. Thus, achieving homogeneity in samples through strategies such as controlling the shape of fungal pellets is a critical step for advanced studies on filamentous fungal chromatin.

Fungal secondary metabolites originate from central metabolic pathways and primary metabolite pools, with acyl-CoAs serving as the critical initial building blocks for generating polyketide and terpene secondary metabolites such as aflatoxin through the catalytic action of PKSs. Amino acids are utilized for the synthesis of non-ribosomal peptide secondary metabolites like penicillin via NRPSs (85). Some secondary metabolites are hybrids synthesized by both enzymes (22). The transcriptional upregulation of these genes under oxidative stress suggests their role in protecting fungi from ROS (86–88). Additionally, many secondary metabolites have been shown to mediate specific interactions between bacteria and fungi, aiding fungi in occupying ecological niches by acting as weapons against bacteria or as signaling molecules that induce prolific sporulation in fungi (22, 89). Here, when interacting with *G. butleri* w5, Skn7 positively regulated the transcriptional levels of *nrps* in *C. cinerea* (Fig. 2). ChIP-seq and EMSA results confirmed the direct binding of Skn7 to the *nrps* promoter (Fig. 4). Furthermore, *easA*, which is involved in the biosynthesis of ergot alkaloids, was also identified as a target of Skn7. Recent studies have reported Skn7’s involvement in aflatoxin biosynthesis in *A. flavus* and triterpenoid biosynthesis in *G. lucidum*, highlighting its critical role in regulating secondary metabolite production in fungi (40, 64). Although metabolomics analysis was not performed in this study, amino acid sequence blasting of proteins involved in the ergot alkaloid pathway from *A. fumigatus* revealed several potential homologs in *C. cinerea*. These included deduced EasF (CC1G_07198, 43% similarity) and EasC (CC1G_09926, 61% similarity), which were predicted to catalyze tryptophan into chanoclavine-I; a deduced EasD (CC1G_02393, 44% similarity) that might transform chanoclavine-I into chanoclavine-I aldehyde; as well as another EasA (CC1G_08222, 55% similarity) and EasG (CC1G_05433, 52% similarity), which were hypothesized to participate in the formation of festuclavine and agroclavine (8, 56). In addition, a deduced EasM (CC1G_04037, 52% similarity) was identified as catalyzing the biosynthesis of fumigaclavines. Furthermore, a deduced CloA (CC1G_11080) exhibiting 51% similarity to the homolog from *Claviceps fusiformis* and a deduced LpsB (CC1G_03009, 45% similarity) were also obtained, both contributing to the production of various lysergic acid derivatives. In the future, metabolomics analysis is necessary to identify the characteristic secondary metabolites in *C. cinerea* during fungal interactions and unravel their specific functions.

**Fig 7:**
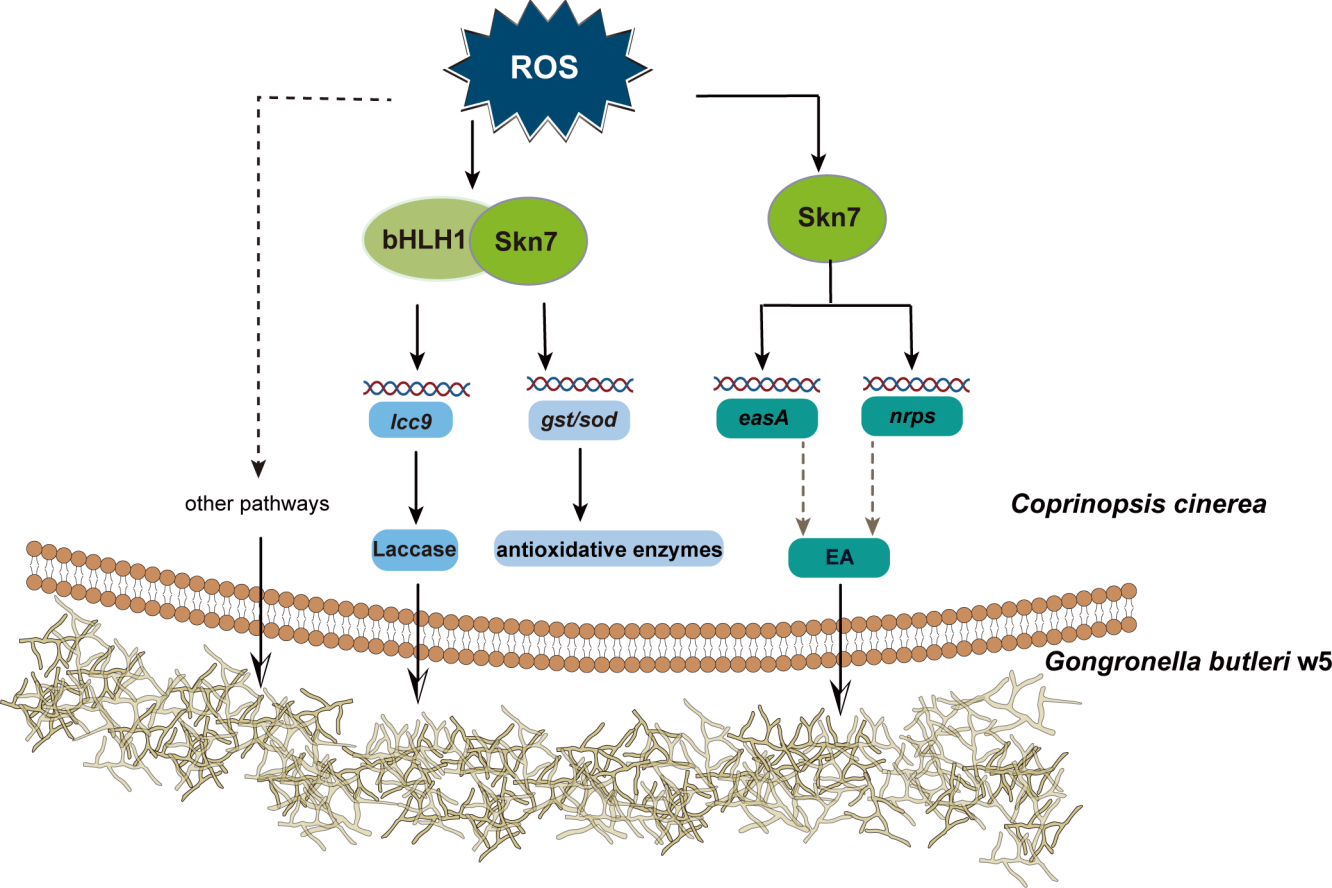
*C*. *cinerea* Skn7 responds to interspecific interaction signals from *G. butleri* w5 and functions in defense against antagonism stress. Skn7 not only orchestrates the expression of Lcc9 and intracellular antioxidative enzymes in collaboration with a novel identified transcription factor, bHLH1, but also regulates the transcription of secondary metabolite biosynthesis genes.

In summary, this study demonstrates that *C. cinerea* Skn7 responds to interspecific interaction signals from *G. butleri* w5 and orchestrates the expression of extracellular enzyme Lcc9 and intracellular antioxidative enzymes in collaboration with a novel identified transcription factor bHLH1 (Fig. 7). The intracellular enzymes are differentially regulated, with increased GST playing a crucial role. Furthermore, Skn7 is also involved in the regulation of secondary metabolite biosynthesis. It directly binds to specific DNA motifs in the promoter region of target genes. These comprehensive strategies enable *C. cinerea* to effectively confront fungal antagonism stress and promote mycelial growth during fungal-fungal interactions.

## MATERIALS AND METHODS

### Strains and cultural conditions

*C. cinerea* Okayama 7 (#130; *A43*, *B43*, *ade8*; ATCC No. MYA-4618) and *G. butleri* w5 (China Center for Type Culture Collection No. AF2012004) were maintained on YMG agar (yeast malt glucose; per liter, 4 g yeast extract, 10 g malt extract, 4 g glucose, and 15 g agar) or PDA (potato dextrose agar; per liter, filtrate of 200 g boiled potato, 20 g glucose, and 15 g agar) plates at 4°C according to reference 90. The vitality of the two fungi was verified by culturing them on plates at 37°C and 28°C, respectively.

The liquid cocultivation of *C. cinerea* and *G. butleri* w5 was conducted as previously (19, 20). SAHX medium (sucrose DL-asparagine HX; per liter, 15 g sucrose, 1.5 g DL-asparagine, 1.0 g KH_2_PO_4_, 0.5 g MgSO_4_·7H_2_O, 0.1 g Na_2_HPO_4_·5H_2_O, 10.0 g CaCl_2_, 1.0 mg FeSO_4_·7H_2_O, 28.0 mg adenine, 2.0 mg CuSO_4_·5H_2_O, and 50.0 µg vitamin B_1_) was used. The WT, Mock, gene-silenced, or gene-overexpressed *C. cinerea* was always first inoculated and cultivated for 36 h before the addition of homogenized *G. butleri* w5 mycelium (2.5%, vol/vol). The latter was sealed in dialysis tubes (molecular mass cutoff of 50 kDa), which were immersed in flasks to avoid direct mycelial contact between the two species. The time 0 h of cocultivation refers to the start of coculture.

For coculture assays on plates, WT, gene-silenced, or gene-overexpressed *C. cinerea,* as well as *G. butleri* w5, were inoculated with mycelial plugs (5 mm in diameter) onto solid SAHX medium. The initial distance between the inocula of *C. cinerea* and *G. butleri* w5 on plates was about 5 cm. Axenic culture of *C. cinerea* was performed by inoculating mycelial plugs onto a solid FAHX medium (in which fructose was substituted for sucrose of SAHX). Colonies were photographed every 24 h. The colony areas were calculated using MATLAB software (MathWorks Inc., MA, USA) by measuring the pixel scale derived from the transformed pictures.

### Construction of *skn7*-silenced, *skn7*-overexpressed, and *bHLH1*-silenced *C. cinerea* transformants

The pYSK7-an*skn7* antisense vector and pYSK7-ov*skn7* vector were ligated with a reverse complementary sequence of 417 bp from *C. cinerea skn7* partial cDNA (bp +546 to +962) and the 1,341-bp full-length cDNA of *skn7*, respectively (primers are listed in Table S2). Similarly, a *bHLH1* antisense silencing fragment comprising the gene sequence of bp +1,876 to +2,189 of *bHLH1* was amplified using primers listed in Table S2 and inserted into plasmid pYSK7 to generate pYSK7-an*bHLH1* antisense vector. The vector construction, *C. cinerea* protoplast preparation, transformation, and subsequent transformant screening procedures were performed according to references 91 and 19. Positive transformants with the selection vector p*Cc*Ade8 were used as the Mock control.

### Quantitative RT-PCR analysis

The Mock, gene-silenced, or gene-overexpressed *C. cinerea* was cocultured with *G. butleri* w5 in liquid SAHX medium for the indicated durations. The mycelia of *C. cinerea* were withdrawn and extracted for total RNA with RNAiso Plus reagent (Takara, Japan), and genomic DNA was removed by using the Evo M-MLV RT Kit (Accurate Biology Co., LTD, China). The SYBR Green Premix Pro Taq HS qPCR Kit (Accurate Biology) and specific PCR primers (Table S2) were utilized to perform fluorescence quantitative reaction on the LightCycler 96 system (Roche, Switzerland). *β*-Actin was used as the internal reference, and the 2*^-ΔΔCT^* method was used to calculate the relative transcriptional levels of *skn7*, *lcc9*, *lcc1*, *lcc5*, *cat*, *sod1*, *sod2*, *trx*, *gst, easA,* and *nrps* (92).

### Determination of intracellular ROS and H_2_O_2_

The fluorogenic probe 2,7-dichlorodihydrofluorescein diacetate (DCFH-DA) (Beyotime Biotech, Shanghai, China) was prepared by diluting it with sterile water to achieve a final concentration of 10 µM. The mycelia of *C. cinerea* cocultured with *G. butleri* w5 in liquid SAHX medium were harvested via centrifugation at indicated time points and subsequently incubated in the diluted DCFH-DA solution at 37°C for 20 min to allow production of DCF dependent on ROS. Following this, the mycelia were washed three times with ddH_2_O, resuspended in 1 mL of sterile water, ground into a powder with glass beads in a freezing grinder (Shanghai Jingxin Co., Ltd, China), and then centrifuged at 12,000 *g* for 5 min. A volume of 200 µL of supernatant was transferred to 96-well plates (Thermo Fisher, Waltham, MA, USA) for fluorescence absorbance detection, with an excitation wavelength of 488 nm and an emission wavelength of 525 nm. All procedures were performed in the dark and in triplicate.

For the detection of intracellular H_2_O_2_, an H_2_O_2_ assay kit (Beyotime Biotech) was used based on a peroxidase-coupled colorimetric method, in which Fe^2+^ is oxidized into Fe^3+^ by H_2_O_2_, which further binds to xylenol orange to form a purple complex. The cocultured mycelia of *C. cinerea* in liquid SAHX medium were harvested at indicated time points, washed three times with ddH_2_O, and subsequently incubated in lysis buffer. The samples were then ground into powder with glass beads in the freezing grinder. Following centrifugation at 12,000 *g* for 5 min, 50 µL of the supernatant was transferred to 96-well plates. To each well, 100 µL of the H_2_O_2_ detection reagent was added and gently mixed, and the fluorescent absorbance was measured at a wavelength of 560 nm. A standard curve was made simultaneously according to the manufacturer’s instructions. The intracellular concentrations of H_2_O_2_ were calculated based on the fluorescent absorbance values obtained from the standard solution with serial dilutions. The experiments were conducted in triplicate.

### Determination of the activities of SOD, CAT, and GST

The enzyme activities of SOD, CAT, and GST in Mock, *skn7*-silenced, or *skn7*-overexpressed *C. cinerea* cells in liquid cocultivation were determined using commercial kits (Sangon Biotech, Shanghai, China) according to the manufacturer’s protocols, each of which is based on a xanthine oxidase-mediated nitroblue tetrazolium reduction reaction system, the UV absorption changes due to the decomposition of H_2_O_2_, and the conjugation of reduced GSH with 1-chloro-2,4-dinitrobenzene (CDNB) to form a GS-DNB adduct, respectively. Briefly, mycelia were harvested, homogenized with the extract solutions provided in the kits, and centrifuged at 8,000 *g* for 10 min. The supernatants were measured by recording the absorbance values at 560, 240, and 340 nm, respectively. All samples were analyzed in triplicate.

### Laccase activity determination and native-PAGE analysis

The laccase activity of the supernatant from *C. cinerea* strains (Mock, gene silenced, or gene overexpressed) cocultured with *G. butleri* w5 was determined every 12 h, using 0.5 mM 2,2′-azino-bis(3-ethylbenzothiazoline-6-sulfonate) (ABTS) as the substrate, generating the stable radical cation ABTS•+ as the colored product with an absorption maximum at 420 nm (93). The same samples were subjected to evaluation using 12% polyacrylamide gels, followed by staining with 1.0 mM ABTS dissolved in citrate-phosphate buffer (pH 4.0) for 10 min. The activity assays and native-PAGE analyses were normalized based on the equivalent wet biomass across all samples.

### Transcriptome analysis

The transformants Mock, OE-1, and R-1 were cocultured with *G. butleri* w5 for 24 h, with three replicates set up for each experiment. Mycelia were harvested, and RNA extraction was performed using TRIzol Reagent (Invitrogen, Carlsbad, CA, USA). cDNA libraries were constructed using the NEBNext Ultra RNA Library Prep Kit following the manufacturer’s protocol and subsequently sequenced on the Illumina NovaSeq 6000 platform (Shanghai Personal Biotechnology Co. Ltd, China).

The clean reads obtained from the raw sequencing data were mapped to the reference genes of *C. cinerea* Okayama 7 (GenBank accession No. AACS00000000), as previously reported (19). The differentially expressed genes among groups were identified using DEGseq version 1.42.0 with |log_2_FoldChange| > 1 and a *P*-value < 0.05. Kyoto Encyclopedia of Genes and Genomes enrichment analysis of the DEGs was performed using the KEGG databases.

### Molecular docking simulations

The three-dimensional structure was simulated using the AlphaFold3 online service (https://golgi.sandbox.google.com) (94). The promoter regions (bp −1,000 to −1) of *gst*, *sod2*, *lcc9*, *easA*, and *nrps* were initially docked with Skn7 to generate the five most probable models, respectively. Subsequently, the models incorporating motifs identified from ChIP-seq analysis were chosen, and the DNA fragments (approximately 30 bp in length) were re-docked with Skn7 using AlphaFold3. The resulting models were visualized using Pymol software (version 2.5.2).

The interaction between full-length Skn7 and bHLH1 was analyzed using the HDOCK Server (http://hdock.phys.hust.edu.cn/). This docking simulation employed a hybrid algorithm that integrates template-based modeling with *ab initio*-free docking (95). The simulated results were visualized using Pymol software (version 2.5.2). The domain of bHLH1 involved in the interaction was designed as a truncated protein.

### Expression and purification of Skn7 and truncated bHLH1 proteins

The DBD cDNA fragment (bp +640 to +1,329) and full-length cDNA fragment of Skn7 were amplified by using specific primers listed in Table S2 and inserted into pET-28a-SUMO (+) vector and pGEX-6P-1 vector, respectively. The cDNA fragment of truncated bHLH1 (bp +931 to +1,470), which was simulated to interact with Skn7, was amplified and inserted into the pET-22b vector. The recombinant vectors, as well as the pGEX-6P-1 vector, were transformed into *Escherichia coli* BL21(DE3) cells, respectively. The positive transformants were induced with 0.5 mM IPTG at 16°C for protein expression. The cells were harvested and lysed by sonication in cold Tris-HCl buffer (50 mM Tris and 500 mM NaCl buffer, pH 7.3). Following centrifugation at 12,000 *g* for 20 min, the supernatant was subjected to affinity chromatography on Ni^2+^-NTA Resin (Novagen, Darmstadt, Germany) or GST Resin (TransGen Biotech, Beijing, China) to obtain purified proteins, including His-SUMO-Skn7 DBD, GST-full-length Skn7, His-truncated bHLH1, and GST. The first two proteins were further digested with enzyme Ulp1 (Cusabio, Wuhan, China) or Thrombin (Roche, Basel, Switzerland) to remove the His-SUMO tag or GST tag, respectively. The protein purity and concentrations were measured using the SDS-PAGE and Bradford methods (Bio-Rad, USA), respectively.

### EMSA

The probes used included DNA fragments from the promoter regions of *gst* (bp −732 to −716), *sod2* (bp −33 to −14), *lcc9* (bp −187 to −173), *easA* (bp −42 to −25), and *nrps* (bp −429 to −410), and five mutant DNA fragments. All probes were labeled with the 6-carboxyfluorescein at the 5′-terminus by Sangon Biotech. The reaction mixtures consisted of 200 ng probes and varying concentrations of purified Skn7 DBD protein, all dissolved in the binding buffer (25 mM HEPES, 4 mM DTT, 100 mM KCl, 2 mM MgCl_2_, 10% glycerol, and 0.01 mg/mL BSA, pH 7.3) to a final volume of 20 µL. Non-labeled probes, added at a 10-fold excess, served as competitive cold probes. Additionally, 200 ng poly d(I-C) was included as a non-specific competitor in each assay. The results were visualized in the gels using a chemiluminescence imager (Smart Chemi 610; Sagecreation, Beijing, China).

### ITC assay

ITC experiments were performed to investigate the interactions between full-length Skn7 and truncated bHLH1 using the iTC200 microcalorimetry system (GE Healthcare) at 25°C. These two proteins were diluted in a Tris buffer (50 mM Tris and 150 mM NaCl, pH 7.3) to final concentrations of 2 µM for Skn7 and 20 µM for truncated bHLH1. Truncated bHLH1 was titrated into Skn7. Each titration started with an initial injection of 0.4 µL, followed by 20 sequential injections of 2 µL, each spaced 5 s apart.

### GST pull down and western blot

The purified GST-full-length Skn7 and His-truncated bHLH1 proteins were used to investigate the interaction between Skn7 and bHLH1. GST-full-length Skn7 protein was incubated with GST resin at 4°C for 1 h, where GST protein was used as a control. After washing three times, His-truncated bHLH1 protein was added to the resin and incubated for 3 h. Following thorough washing, the resins were thermally denatured at 95°C for 5 min with a 5× SDS loading buffer. The samples were subjected to electrophoresis on 12% SDS-PAGE gels and subsequently transferred onto the PVDF membranes (Merck, NJ, USA) using the Thermo Scientific Owl VEP-2 system. The PVDF membranes were blocked with a 5% solution of non-fat dried milk and then incubated with an anti-His antibody (Abcam, Cambridge, Britain; diluted 1:1,000), followed by incubation with the horseradish peroxidase goat anti-rabbit IgG (H + L) (Abcam; diluted 1:5,000). Finally, autoradiography was performed using the BeyoECL Plus Kit (Beyotime Biotech).

### Skn7-specific antibody preparation and Co-IP mass spectrum

The purified full-length Skn7 protein was injected into New Zealand White rabbits to prepare a specific antibody against Skn7 (anti-Skn7) by the GenScript Company (Nanjing, China).

The *C. cinerea* mycelia cocultured with *G. butleri* w5 were harvested, washed, and used for nuclear protein extraction as previously described (96). The nuclear protein was incubated with protein A-Dynabeads (Invitrogen, Vilnius, Lithuania) coupled with an anti-Skn7 antibody. Rabbit IgG was used as the negative control. After washing five times, the bound proteins were subjected to LC-MS/MS detection (Applied Protein Technology, Shanghai, China). The MS data were analyzed by MaxQuant software (version 1.5.3.17). Proteins were identified by searching the data against the annotated genome of *C. cinerea* Okayama 7 (GenBank accession No. AACS00000000).

### Chromatin immunoprecipitation followed by massively parallel sequencing

Mycelia of the WT parent *C. cinerea* were harvested after 24 h of cocultivation. They were incubated with 1% formaldehyde at 37°C for 10 min to induce protein-DNA cross-linking, followed by the addition of glycine to a final concentration of 125 mM to terminate the reaction. Subsequently, the mycelia were lysed and homogenized with glass beads in the ChIP lysis buffer (50 mM HEPES, 137 mM NaCl, 1 mM EDTA, 1% Triton X-100, 0.1% deoxycholic acid, and 0.1% SDS, pH 7.5) using a freezing grinder. After centrifugation at 4°C, the supernatant was collected and sonicated to yield fragmented nucleic acids ranging from 200 to 1,000 bp. Approximately 1 mg of chromatin was incubated overnight at 4°C with 10 µg of anti-Skn7 antibody or rabbit IgG (Thermo Fisher). Protein G-Dynabeads (Thermo Fisher) were pre-blocked with bovine serum albumin and subsequently added to the mixture, which was then rotated at 4°C for an additional 6 h. The protein-DNA fragment complexes were eluted from the beads and reverse cross-linked. Finally, the protein was degraded by proteinase K, and the DNA fragments were extracted. The samples were sent to Shanghai Personal Biotechnology Co., Ltd for sequencing.

The DNA quality was confirmed by analyzing in an Agilent 2100 Bioanalyzer. Libraries were constructed using the KAPA Hyper Prep Kit for Illumina (#KK8504) and sequenced on the Illumina NovaSeq Xplus platform. FastQC (http://www.bioinformatics.babraham.ac.uk/projects/fastqc) was used to evaluate the quality of the raw sequencing reads from three independent ChIP-seq experiments. Cutadapt version 1.2.1 was used to yield high-quality clean data (97). The clean reads were aligned to the *C. cinerea* genome using Bowtie2 software (version 2.2.6). MACS3 software (version 3.0.0a7) was employed for peak calling (to identify significantly enriched peaks at *q* < 0.05) on individual replicates for each ChIP and input pair. Differential peak analysis was conducted using MEME-ChIP (http://meme-suite.org/tools/meme-chip) (98, 99).

### Statistical analysis

All the experiments were performed independently in triplicate. GraphPad Prism (version 9.0) was used for statistical analysis of experimental data by one-way ANOVA and Student’s *t*-test, and the significance level was set at 0.05 (*P* ≤ 0.05, *P* ≤ 0.01, *P* ≤ 0.001, or *P* ≤ 0.0001) among samples.

## Data Availability

The transcriptomic and ChIP-seq data are publicly available in the National Centre for Biotechnology Information (NCBI): BioProject SRA ID: PRJNA1212308 and PRJNA1212327. The data sets generated and analyzed during the present study are available and included either within this article or in the supplemental material. The rRNA gene sequences (5.8S and 18S) of *Gongronella butleri* w5 are available in GenBank under accession numbers PV116320 (5.8S), and PV116318 (18S).

## SUPPLEMENTAL MATERIAL

Figure S1 to S8 and Table S2 spectrum.00484-25-s0001.docx Summary selected experimental results in this study and primers used in this study.

Table S1 spectrum.00484-25-s0002.xlsx Summary of omics Data and selected experimental results in this study.

ASM does not own the copyrights to Supplemental Material that may be linked to, or accessed through, an article. The authors have granted ASM a non-exclusive, world-wide license to publish the Supplemental Material files. Please contact the corresponding author directly for reuse.
