# Supplementary material for: The regulatory network of Coprinopsis cinerea transcription factor Skn7 collaborates with bHLH1 during fungal-fungal interactions
Source: Microbiol Spectr. 2025 Jul 30;13(9):e00484-25. doi: 10.1128/spectrum.00484-25 (PMC12403569; doi:10.1128/spectrum.00484-25)
Supplement: Figure S1 to S8 and Table S2 — Summary selected experimental results in this study and primers used in this study. [file spectrum.00484-25-s0001.docx]

**The regulatory network of *Coprinopsis cinerea* transcription factor Skn7 collaborative with bHLH1 during fungal-fungal interactions**

Huifang Zhao^1,2,3^, Na Pang^1,2,3^, Xinyue Meng^1,2,3^, Qiuyu Qian^1,2,3^, Qiqi Han^1,2,3^, Qun Han^1,2,3^, Xinlei Zhang^1,2,3^, Zemin Fang^1,2,3*^, Juanjuan Liu^1,2,3*^

^1^ School of Life Sciences, Anhui University, Hefei 230601, Anhui, China

^2^ Anhui Key Laboratory of Biocatalysis and Modern Biomanufacturing, Hefei 230601, Anhui, China

^3^ Anhui Provincial Engineering Technology Research Center of Microorganisms and Biocatalysis, Hefei 230601, Anhui, China

*Corresponding authors

Phone/Fax: +86-551-63861861

Email: zemin_fang@ahu.edu.cn; liu_juan825@ahu.edu.cn (to LJ).

**
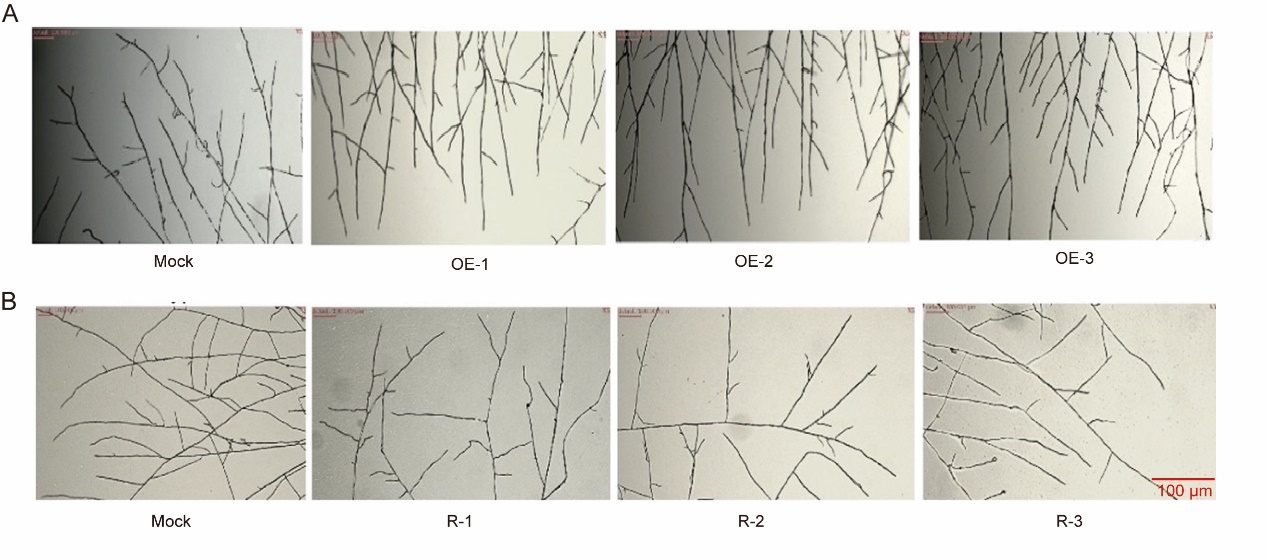
**

**Fig. S1** **Effects of *skn7* overexpression (A) and silencing (B) on the hyphal branches of *C. cinerea* during interspecific interaction with *G. butleri* w5 on plates.** Scale bar, 100 µm.

**
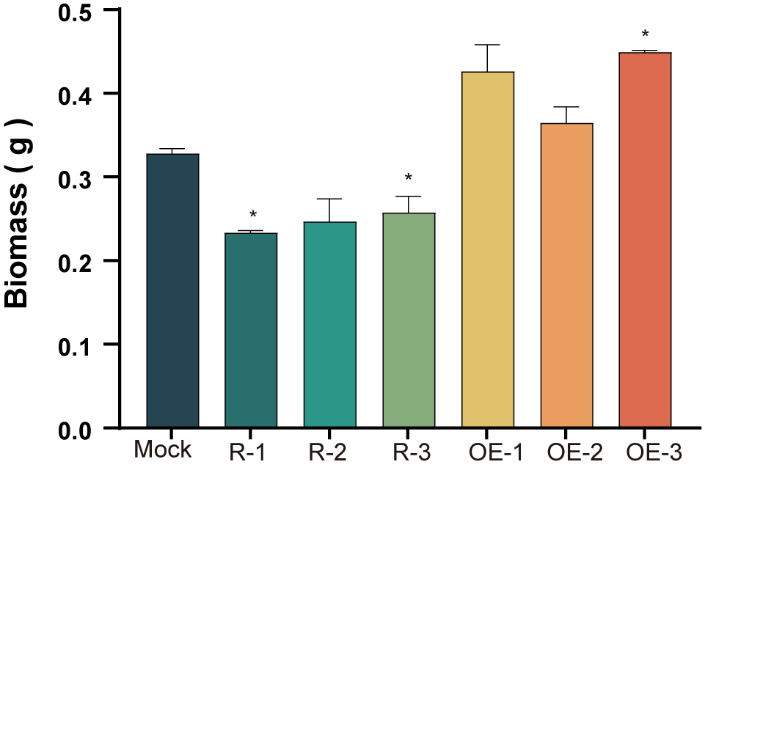
**

**Fig. S2** **The biomasses of *skn7*-overexpressed and *skn7*-silenced *C. cinerea* when cocultured with *G. butleri* w5 for 48 h in liquid culture.** Data show mean ± standard deviation, n = 3. ** *P* < 0.01.

**
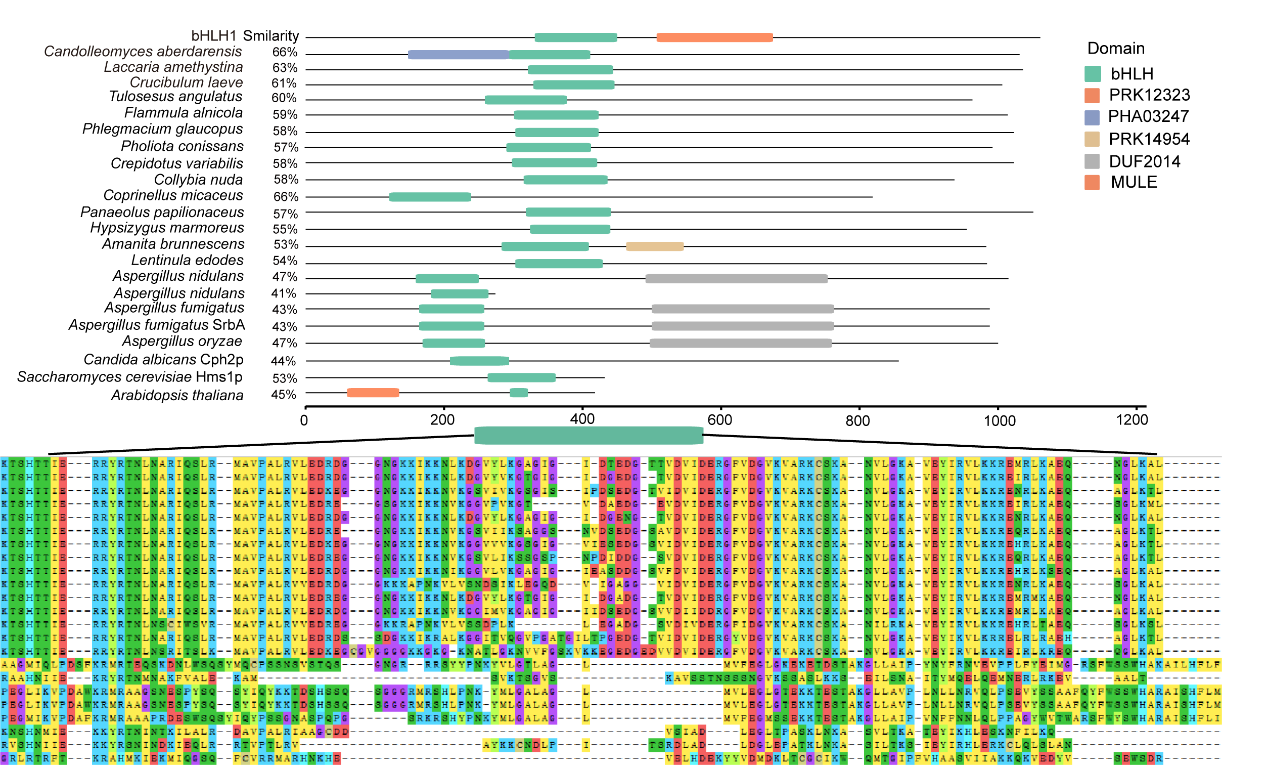
**

**Fig. S3 Amino acid sequence alignment and structural domain prediction of bHLH1 and homologs from other species.** Sequences include *Candolleomyces aberdarensis* RXM16841.1, *Laccaria amethystina* LaAM-08-1 KIJ99079.1, *Crucibulum leave* TFK41459.1, *Tulosesus angulatus* KAF6744872.1, *Flammula alnicola* KAF8970448.1, *Phlegmacium glaucopus* KAF8804632.1, *Pholiota conissans* KAF9486247.1, *Crepidotus variabilis* KAF9527446.1, *Collybia nuda* KAF9457138.1, *Coprinellus micaceus* TEB19201.1, *Panaeolus papilionaceus* KAF99057131.1, *Hypsizygus marmoreus* RDB30417.1, *Amanita brunnescens Koide* BX004 KAF9688856.1, *Lentinula edodes* KAJ3913799, *Aspergillus nidulans* FGSC A4 XP680930 and EAA61422.1, *Aspergillus fumigatus* KEY82192.1 and SrbA QRK14818.1, *Aspergillus oryzae* 3.042 EIT80526.1, *Candida albicans* Cph2p XP_712305.1, *Saccharomyces cerevisiae* Hms1p KAJ1546992.1, and *Arabidopsis thaliana* CAD5332832.1.

**
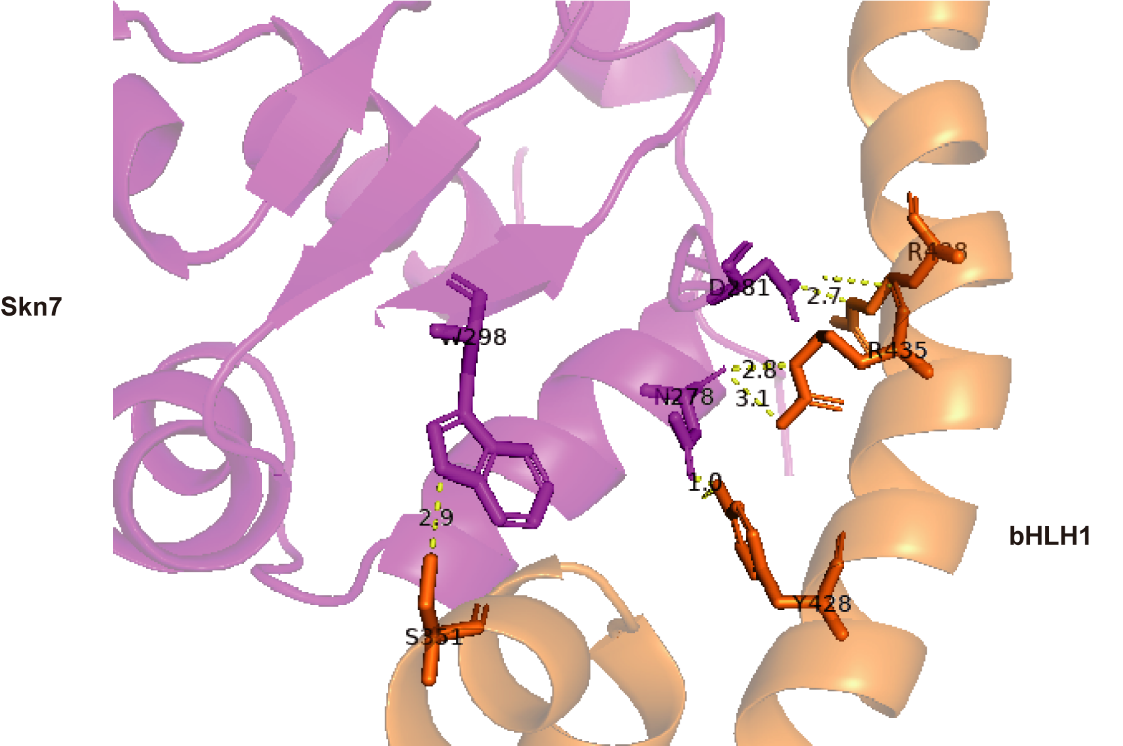
**

**Fig. S4 HDOCK analysis shows direct binding between Skn7 and bHLH1.**

**
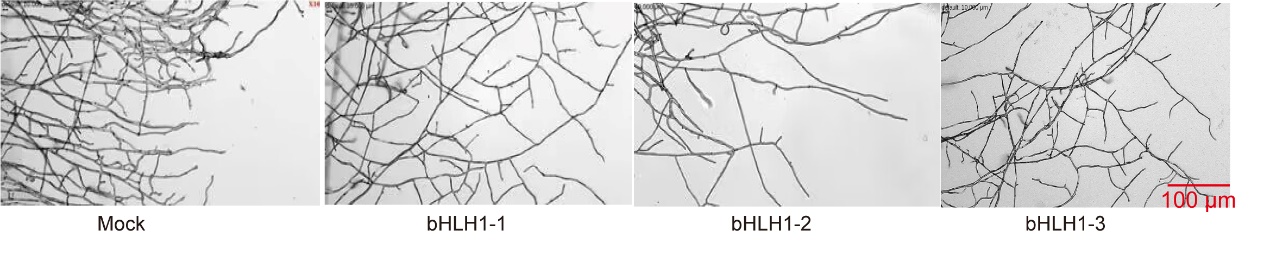
**

**Fig. S5** **Effects of *bHLH1* silencing on the hyphal branches of *C. cinerea* during interspecific interaction with *G. butleri* w5 on plates.** Scale bar, 100 µm.

**
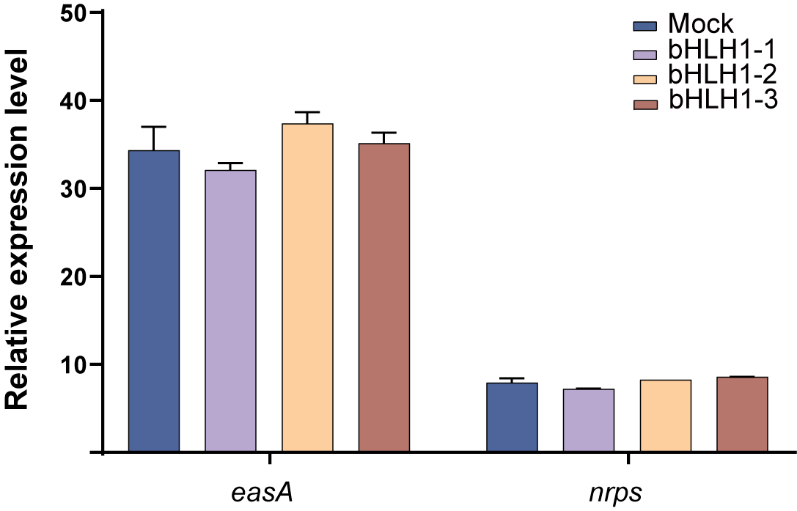
**

**Fig. S6 qRT-PCR analysis of the transcriptional levels of *nrps* and *easA* in Mock and *bHLH1*-silenced *C. cinerea* at 36 h of liquid coculture.** Data show mean ± standard deviation, n = 3.

**
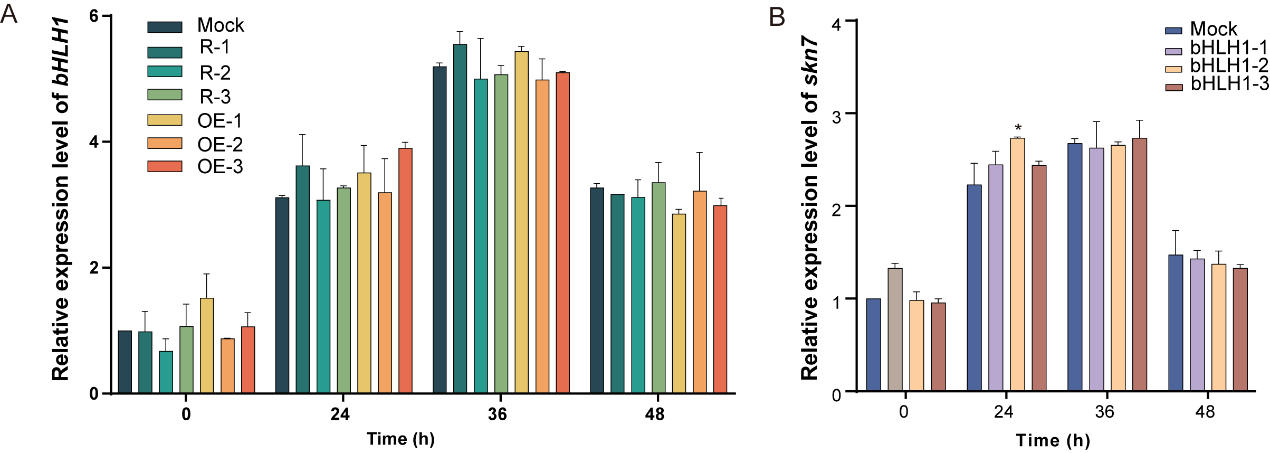
**

**Fig. S7 Skn7 and bHLH1 function independently of each other. (A)** The transcriptional levels of *bHLH1* in *skn7*-overexpressed and -silenced transformants. **(B)** The transcriptional levels of *skn7* in the Mock and *bHLH1*-silenced transformants. Data show mean ± standard deviation, n = 3.

**
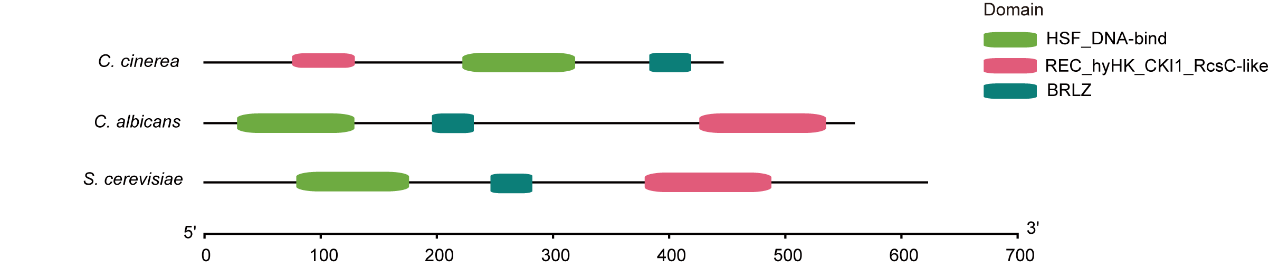
**

**Fig. S8** Schematic representation of Skn7 in *C. cinerea*, *C. albicans* and *S. cerevisiae.*

**Table S1 Summary of omics Data and selected experimental results in this study**

Table S1-1 ROS concentrations, transcriptional levels and enzymatic activities of laccase, CAT, SOD, GST in the Mock, *skn7*-overexpressed and -silenced transformants during submerged coculture

Table S1-2 DEGs in transformants R-1

Table S1-3 DEGs in transformants OE-1

Table S1-4 Additional data supporting Figure 2

Table S1-5 Statistics of ChIP peak information

Table S1-6 Proteins identified by LC-MS/MS

Table S1-7 Summary of ChIP peak annotations

**Table S2 Primers used in this study**

| **Name** | **Sequence (5’-3’)** | **Purpose** |
| --- | --- | --- |
| R*-skn7*-F | CTCCCATCTACACACAACAAGCTTATCGCCTGAACAGCCGGGGTAGAATGG | Cloning of *skn7* antisense fragment |
| R*-skn7*-R | CACTGGCCCTCTGGTCAACTATAATATTATCACTTGGCAAGCGGACCAGAG | Cloning of *skn7* antisense fragment |
| *skn7*-overexpression-F | CTCCCATCTACACACAACAAGCTTATCGCCATGGTGAGCAAGGGCGAGGAG | Cloning full-length cDNA of *skn7* |
| *skn7*-overexpression-R | AGATCCACTACCACTACCTGAACCTCACTTGTACAGCTCGTCCATGCCG | Cloning full-length cDNA of *skn7* |
| R-*bHLH1*-1F | \| CGTCAAACCCAACCAACTCC \| \| --- \| | Cloning of *bHLH1* antisense fragment |
| R-*bHLH1*-1R | CCTCCCAATGTCCAACCTCA | Cloning of *bHLH1* antisense fragment |
| Skn7-DBD-1-F | AACGACGATCCTATGCCCCA | Cloning of *skn7*-DBD fragment |
| Skn7-DBD-1-R | CTGCATCAACCCATCCTGCT | Cloning of Skn7-DBD fragment |
| bHLH1-TP-F | GGTGGACAGCAAATGGGTCGCGGATCCACTCCGACCAATGCTATGC | Cloning of truncated bHLH1 fragment |
| bHLH1-TP-R | TCAGTGGTGGTGGTGGTGGTGCTCGAGTTCACGCCAGCGCTGTT | Cloning of truncated bHLH1 fragment |
| qRT-*skn7*-F | TGTACAAGATGCTCGAAGACCCA | qRT-PCR of *skn7* |
| qRT-*skn7*-R | TTCATGTCCTTTACGACGAAACAGTC | qRT-PCR of *skn7* |
| qRT-*bHLH1*-F | TTTGTGGAGTTGGTTGGGTTTG | qRT-PCR of *bHLH1* |
| qRT*-bHLH1-R* | GGAGGTATGGATGGCGAGGG | qRT-PCR of *bHLH1* |
| qRT*-β-actin-F* | CTCTGGAGTTATGGTAGGAATGGGC | qRT-PCR of *β-actin* |
| qRT*-β-actin-R* | GATGCCATGTTCGATGGGGTACTTG | qRT-PCR of *β-actin* |
| qRT-cat-F | AGGGCTCCAGAGGAAGTGCTGATTG | qRT-PCR of *cat* |
| qRT*-cat-R* | TGAACGAAATCGGGGAACTTGATGGCA | qRT-PCR of *cat* |
| qRT-*gpx*-F | CTTCGTGGCAAGGTTGTGTTGATTT | qRT-PCR of *gpx* |
| qRT-*gpx*-R | ACTTCGTTGGTGTTGTCTCCGTTCA | qRT-PCR of *gpx* |
| qRT-*sod2*-F | CCTTGAAGTTCAACGGAGGAGGCCACAT | qRT-PCR of *sod2* |
| qRT*-sod2*-R | TTCCTTGAGGACACCACCGTTGCCCTTG | qRT-PCR of *sod2* |
| qRT-*sod1*-F | CACTGGGACCGCCTCGGGTATCGTA | qRT-PCR of *sod1* |
| qRT-*sod1*-R | GCTTGGTGAACATGGAATCCACGCAG | qRT-PCR of *sod1* |
| qRT*-trx*-R | CGAGATATTCAAAGAACTGGCGGGA | qRT-PCR of *trx* |
| qRT-*trx*-R | TCCTCGGAGTAGCAAAAAGGTCGGA | qRT-PCR of *trx* |
| qRT-*lcc1*-F | ATGTTCAAGAACCTCCTCTCGT | qRT-PCR of *lcc1* |
| qRT*-lcc1*-R | ACGTTCGCGTTGGTGAGGGTCAT | qRT-PCR of *lcc1* |
| qRT-lcc5-F | ATGTCGTTTGCTTGGAAAGCAT | qRT-PCR of *lcc5* |
| qRT-*lcc5*-R | TGGTCATAGTATCTTGGTTGCCAAT | qRT-PCR of *lcc5* |
| qRT*-lcc9*-F | ATGTCCAGGAAACTTTTCTCTCTCG | qRT-PCR of *lcc9* |
| qRT-*lcc9*-R | ATGTTCGAGACCGTCATGGTACT | qRT-PCR of *lcc9* |
| qRT-*easA*-F | CGATTACGTGGCTTTGAGG | qRT-PCR of *easA* |
| qRT-*easA*-R | GGAACTAAGGAGTTCAGCGATA | qRT-PCR of *easA* |
| qRT-*nrps*-F | CCAAGCCTCGCGATTTTACT | qRT-PCR of *nrps* |
| qRT-*nrps*-R | CACCGTTGACCATAGAGCCA | qRT-PCR of *nrps* |
| qRT-*gst*-F | ATCTGCCTTCCGCAATACCA | qRT-PCR of *gst* |
| qRT-*gst* -R | AGGATCTGTCCCAGGATAGTCG | qRT-PCR of *gst* |
